# UNVEILING ASSOCIATIONS BETWEEN THE WORK-RELATED AND LEISURE-TIME PHYSICAL ACTIVITY WITH BIOLOGICAL AGE

**DOI:** 10.1093/geroni/igae098.0242

**Published:** 2024-12-31

**Authors:** Mingyue Hu

**Affiliations:** Central South University, Changsha, Hunan, China (People’s Republic)

## Abstract

**Objectives:**

This study aimed to examine the association and dose-relationship between different intensities of work-related and leisure-time physical activity (PA) and biological age (BA). Design: A cross-sectional study. Setting and participants: We used data of 5,216 participants aged 45 and over in the National Health and Nutrition Examination Surveys (NHANES). Measurements: PA was self-reported using the Global Physical Activity Questionnaire (GPAQ), and BAs were calculated by the PhenoAge and Klemera-Doubal method (KDM). In the analyses, survey-weighted generalized linear models were used to explore associations between PA and BAs. Restricted cubic spline characterized the dose-response relationship of PA and BAs. Subgroup analyses were conducted by age, sex, and ethnicity.

**Results:**

Work-related light-intensity PA (LPA) was significantly associated with younger PhenoAge (β=0.507, 95%CI: -0.957, -0.056, p = 0.031) and KDM (β=0.908, 95%CI: -1.774, -0.042, p = 0.042). There was a U-shaped dose-response relationship between LPA and BAs (all p < 0.05). Leisure-time moderate-intensity PA (MPA) was associated with younger PhenoAge (β=0.789, 95%CI: -1.573, -0.006, p=0.049) and KDM (β=1.910, 95%CI: -3.417, -0.402, p = 0.017). There was a U-shaped dose-response relationship between MPA and BAs (all p < 0.05). In subgroup analyses for older adults, all intensities of PA were significantly associated with decreased BAs (all p < 0.05). Conclusions: Doing PA could delay the BAs, specifically the work-related LPA and leisure-time MPA. Older adults can benefit from all intensities of PA regardless of the PA of different environments and intensities.

